# A case of recurrent, bilateral ovarian mature teratoma in a young woman

**DOI:** 10.1186/1472-6874-14-57

**Published:** 2014-04-13

**Authors:** Che-Fu Chang, Chen-Kuo Lin

**Affiliations:** 1 Department of Family Medicine, Taoyuan Armed Forces General Hospital, No.168, Zhongxing Rd., Longtan Township, Taoyuan County, 32551, Taiwan; 2 Department of Obstetrics and Gynecology, Taoyuan Armed Forces General Hospital, No.168, Zhongxing Rd., Longtan Township, Taoyuan County, 32551, Taiwan

**Keywords:** Teratomas, Ovarian, Recurrence, Resection

## Abstract

**Background:**

Ovarian mature cystic teratomas are common, benign, pelvic tumors that are easily detected by pelvic ultrasonography. However, patients with recurrent teratomas are rarely noted, and cases of bilateral teratomas are even less common.

**Case presentation:**

A young woman with a recurrent, right ovarian teratoma had previously undergone surgical removal 2 times. After the second surgery, she underwent regular out-patient follow-up, and no residual tumor was observed. However, 3 years after the second surgery, she developed recurrent, bilateral ovarian teratomas, in conjunction with elevated carbohydrate antigen-125 levels.

**Conclusion:**

Routine checking of the contralateral ovary during the surgical procedure along with frequent postoperative pelvic sonography for both ovaries in the patient at high recurrence rich is necessary. Additionally, the features of that kind tumor may mislead the surgeon into performing more extensive surgery that might compromise the fertility of young patients.

## Background

Mature cystic teratomas, also called dermoid cysts, are a type of germ cell tumor comprising well-differentiated tissues and three germ cell layers: ectoderm, mesoderm, and endoderm [1]. These tumors account for 10–20% of all ovarian neoplasms and have a peak incidence in women aged 20–40–years [1-3]. Mature cystic teratomas are usually benign, but in rare cases (approximately 0.1–0.2%), they may undergo malignant transformation [4]. These tumors are usually slow-growing and most are unilateral [1]; approximately 10% of cases are bilateral. In the present report, we describe a case involving an unusual course for a mature cystic teratoma.

## Case presentation

**Figure 1:**
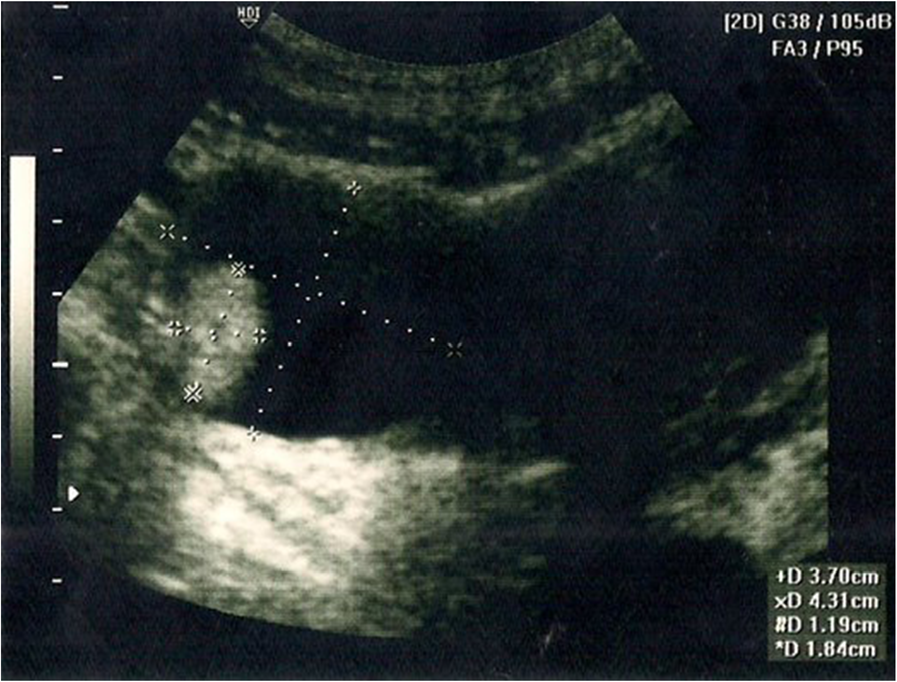
Transabdominal ultrasound revealed a well-defined, hyperechoic mass (4.3 × 3.7 cm) with an appearance resembling that of a teratoma, just adjacent to the uterus.

A 20-year-old nulliparous woman presented to our gynecologic department because of intermittent abdominal pain and a 3-month history of dysmenorrhea. She had a history of 2 right ovarian teratomas that were managed surgically. At the age of 16 years, she experienced lower abdominal pain for 2 weeks. A subsequent pelvic sonography showed a complex, right ovarian cyst, of approximately 5.36 × 4.3 cm in size; her carbohydrate antigen (CA) 125 level was 23.4 U/mL (normal, 0–25 U/mL, at our hospital). We performed a laparoscopically assisted ovarian cystectomy, and the subsequent pathologic analysis revealed a mature cystic teratoma. During the laparoscopy, the left ovary was visually examined, and morbid findings were not noted. One month after the surgery, her symptoms had improved and no residual tumor was observed on pelvic sonography. The patient was subsequently lost to follow-up. After 1 year, she presented to our out-patient department with a 2-month history of prolonged menstrual periods; a right ovarian complex mass, approximately 3.7 × 4.3 cm, with a solid component, was noted during a pelvic ultrasound examination (Figure 1). Her CA125 level was 11.9 U/mL. She, again, underwent laparoscopically assisted ovarian cystectomy in our hospital, and the pathological report revealed another mature cystic teratoma. However, no abnormal findings were noted for the left ovary.

**Figure 2:**
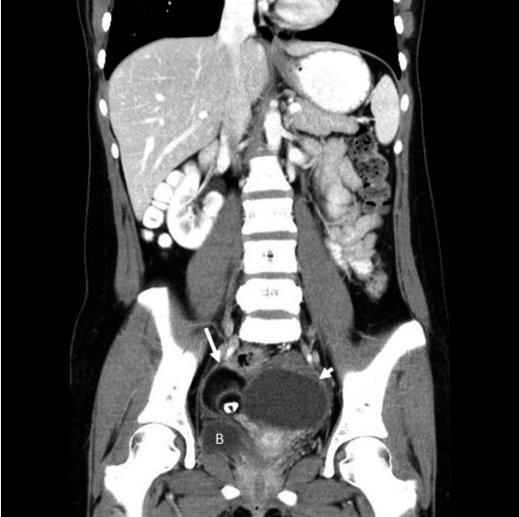
Coronary, post-contrast enhancement imaging shows a large heterogeneous tumor arising from the left adnexa (arrow); in addition, a right adnexal tumor is noted, with a focal radiodense lesion (shorter arrow), consistent with bilateral ovarian complex tumors.

She received regular follow-up care every 6 months and had been well for the next 3 years. However, at the age of 20 years, she presented with intermittent abdominal pain as well as dysmenorrhea that had persisted for several months. A complex mass, that had an approximately 10% solid component, was detected on sonographic examination. In addition, her CA125 level was 103.1 U/mL. Based on the findings of serological tests, we suspected the presence of a malignancy. The patient underwent a contrast-enhanced abdominal and pelvic computed tomography (CT) scan, which revealed an 8.9 × 5.7-cm, complex, cystic tumor of the left adnexa, with compression and displacement of the urinary bladder. Moreover, a 5.1 × 3.9-cm, complex, cystic, partially solid tumor was noted on the right adnexa (Figure 2).

Due to the presence of bilateral adnexal lesions and the suspicion of malignancy, the patient underwent an exploratory laparotomy, bilateral ovarian cystectomy, and pelvic adhesiolysis. During the laparotomy, a capsulated, enlarged, 9 × 6 cm, left ovarian tumor was detected, with abundant sebaceous and hair-containing tissue. The right mass appeared to be a teratoma, 5 × 4 cm in size. Histopathological findings confirmed the presence of bilateral, mature cystic teratomas, composed of sebaceous and keratinous materials as well as hair shaft components. No evidence of malignancy was found. After discharge, the patient underwent follow-up at 1, 3, 9, and 15 months. She did not report abdominal pain or menstrual abnormalities, and a pelvic sonography did not demonstrate any signs of recurrence or residual tumor.

## Discussion

The word “teratoma” is derived from Greek word “teraton” meaning monster and the term “dermoid cyst” was coined by Leblanc in 1831 [1,2]. Teratomas are often composed of multiple embryologic layers and are classified as either mature or immature types. The mature type of teratoma is benign, whereas the immature type of teratoma is also benign but has a more aggressive course [3]. Mature cystic teratomas are the most common type, accounting for approximately 10–20% of the total cases of ovarian tumors [4]. The disease occurs in patients of almost any age, from childhood to post-menopause, although the peak incidence is reported in women aged 20–40 years [5]. Mature cystic teratomas are usually slow-growing, with an estimated growth rate of 1.8 mm/year, [6] although some have been shown to grow more rapidly. Mature cystic teratomas are usually unilateral, with only approximately 8–15% being bilateral; moreover, they have a long-term recurrence rate, following surgical excision, of 4.2% [7]. In the present case, the patient had a history of 3 right ovarian teratomas and the clinical course involved a shift from unilateral to bilateral tumors. To our knowledge, these findings are rare among cases of mature cystic teratomas of the ovary.

The increasing levels of estrogen and progesterone may explain the increase in size of mature cystic teratomas after puberty, and their arrested growth after menopause [8]. In adult patients, mature cystic teratomas are often detected incidentally during routine imaging procedures or during abdominal or pelvic surgeries performed for other reasons; most of these cases (64.5%) are asymptomatic [9]. However, in children and adolescents, these ovarian tumors may also show different clinical manifestations, such as abdominal pain and distension, caused by tumor torsion or ligament irritation [10].

Ultrasonography and tumor markers, such as CA125, CA19-9, and alpha-fetoprotein, are common tools used for the early detection and characterization of ovarian masses, such as mature or immature teratomas. Ultrasonography is an excellent, non-invasive, investigative procedure that can be used for women of any age [11,12]. Among the above mentioned tumor makers, serum CA19-9 is the most reliable biomarker of ovarian mature cystic teratomas; higher levels of serum CA19-9 are correlated with larger tumor sizes. However, the diagnostic value of CA19-9 in patients with mature cystic tumors is low when used alone [13]. Clinically, serum CA125 is still used to distinguish between benign and malignant pelvic masses [14]. In the present case, a shift from a unilateral to bilateral ovarian involvement, with elevated CA125 levels (103.1 U/mL), was noted. Based on the findings of serological examination, we suspected the presence of a malignancy. Therefore, she underwent a contrast-enhanced abdominal and pelvic CT scan, as well as an exploratory laparotomy. The histopathological findings confirmed the presence of bilateral mature cystic teratomas.

For most patients with mature cystic teratomas, laparoscopic or laparotomic surgical excision can provide a definitive diagnosis, afford symptom relief, and prevent complications [15]. Laparoscopic management of ovarian tumors is a potentially safer alternative for young women in whom fertility preservation is a desired outcome [16]. The reported incidence of postsurgical recurrence on the same ovary is 3–4% [17]. Previously, the contralateral ovary was also recommended for biopsy during surgery, but this procedure is no longer indicated due to the availability of accurate sonographic imaging [17]. According to Harada et al., young age (<30 years), large cyst size (diameter, >8 cm), and bilateral occurrence are predictive risk factors for recurrence, with the risk of recurrence being especially high in the presence of more than one of these factors [18]. In the present case, her young age (16 years) at the time of the initial diagnosis was a risk factor for recurrence. However, cases involving two postsurgical recurrences and a shift from unilateral to bilateral ovarian involvement are rare.

## Conclusions

In order to prevent residual disease, routine checking of the contralateral ovary during surgical resection of a recurrent lesion is necessary. To increase the likelihood of detecting recurrent disease, frequent (every 3 months), postoperative pelvic sonographic examinations of both ovaries are also necessary in patients at high risk of recurrence. As suggested by this case, clinicians might be easily misled by the apparent features of these ovarian tumors that may suggest malignancy, resulting in an extensive surgery that might compromise the fertility of young patients.

### Consent

Written consent was obtained from the patient for publication of this study.

## Competing interests

The authors declare that they have no competing interests.

## Authors’ contribution

C-KL: Investigation of the patient, Literature research, Drafting of the manuscript, and corresponding author; C-FC: Literature research, and Drafting of the manuscript. Both authors read and approved the final manuscript.

## Pre-publication history

The pre-publication history for this paper can be accessed here:

http://www.biomedcentral.com/1472-6874/14/57/prepub
